# Supplementary material for: From sequence to enzyme mechanism using multi-label machine learning
Source: BMC Bioinformatics. 2014 May 19;15:150. doi: 10.1186/1471-2105-15-150 (PMC4229970; doi:10.1186/1471-2105-15-150)
Supplement: Additional file 2 — Java code of ml2db. Additional file ml2db_code.tar.gz contains the Java source code to run the multi-label machine learning experiments and save the results to database. The code’s Javadoc is included. [file 1471-2105-15-150-S2.zip › additional file 2/ml2db/utils/doc/cern/colt/PersistentObject.html]

PersistentObject


JavaScript is disabled on your browser.


- Overview
- Package
- Class
- Use
- Tree
- Deprecated
- Index
- Help

- Prev Class
- Next Class

- Frames
- No Frames

- All Classes

- Summary:
- Nested |
- Field |
- Constr |
- Method

- Detail:
- Field |
- Constr |
- Method


cern.colt

## Class PersistentObject

- java.lang.Object
- - cern.colt.PersistentObject

- All Implemented Interfaces:
  :   java.io.Serializable, java.lang.Cloneable

  Direct Known Subclasses:
  :   AbstractDistribution, RandomElement

  ---

    

  ```
  public abstract class PersistentObject
  extends java.lang.Object
  implements java.io.Serializable, java.lang.Cloneable
  ```

  This empty class is the common root for all persistent capable classes. If
  this class inherits from java.lang.Object then all subclasses are
  serializable with the standard Java serialization mechanism. If this class
  inherits from com.objy.db.app.ooObj then all subclasses are
  *additionally* serializable with the Objectivity ODBMS persistance
  mechanism. Thus, by modifying the inheritance of this class the entire tree
  of subclasses can be switched to Objectivity compatibility (and back) with
  minimum effort.

  See Also:
  :   Serialized Form

- - ### Method Summary

    Methods

    | Modifier and Type | Method and Description |
    | --- | --- |
    | `java.lang.Object` | `clone()` Returns a copy of the receiver. |

    - ### Methods inherited from class java.lang.Object

      `equals, getClass, hashCode, notify, notifyAll, toString, wait, wait, wait`

- - ### Method Detail


    - #### clone

      ```
      public java.lang.Object clone()
      ```

      Returns a copy of the receiver. This default implementation does not
      nothing except making the otherwise protected clone method
      public.

      **Overrides:**
      :   `clone` in class `java.lang.Object`

      Returns:
      :   a copy of the receiver.


- Overview
- Package
- Class
- Use
- Tree
- Deprecated
- Index
- Help

- Prev Class
- Next Class

- Frames
- No Frames

- All Classes

- Summary:
- Nested |
- Field |
- Constr |
- Method

- Detail:
- Field |
- Constr |
- Method
